# Mitochondrial Uncoupler, 2,4-Dinitrophenol, Reduces Spinal Cord Paralysis and Retinal Ganglion Cell Loss in the Experimental Autoimmune Encephalomyelitis Model of Multiple Sclerosis

**DOI:** 10.3390/biom15020189

**Published:** 2025-01-28

**Authors:** Nuala O’Neill, Reas S. Khan, Suad Abd Alhadi, Kimberly E. Dine, John G. Geisler, Brahim Chaqour, Ahmara G. Ross, Kenneth S. Shindler

**Affiliations:** 1 Department of Ophthalmology, Scheie Eye Institute, FM Kirby Center for Molecular Ophthalmology, University of Pennsylvania, Philadelphia, PA 19104, USA; mailbox.nuala@gmail.com (N.O.); reaskhan@gmail.com (R.S.K.); suad.abdalhadi@pennmedicine.upenn.edu (S.A.A.); kdine@pennmedicine.upenn.edu (K.E.D.); brahim.chaqour@pennmedicine.upenn.edu (B.C.); ahmara.ross@pennmedicine.upenn.edu (A.G.R.); 2 Mitochon Pharmaceuticals, Inc., 970 Cross Lane, Blue Bell, PA 19087, USA; jgeisler@mitochonpharma.com; 3 Department of Neurology, University of Pennsylvania, Philadelphia, PA 19104, USA

**Keywords:** EAE, optic neuritis, RGC neuroprotection, dinitrophenol, mitochondrial uncoupling agent

## Abstract

Optic neuritis is an inflammatory demyelinating disease of the optic nerve that often occurs in multiple sclerosis (MS) patients. Sixty percent of patients develop some level of permanent visual loss due to retinal ganglion cell (RGC) damage following optic neuritis, with no known treatment to prevent this loss. Prior studies showed that MP201, a prodrug of 2,4-dinitrophenol (DNP) administered in the experimental autoimmune encephalitis (EAE) mouse model of MS attenuated optic neuritis with preserved vision, increased retinal ganglion cell (RGC) survival, decreased axon loss, and reduced demyelination. Oral administration of MP201, which converts to active form DNP after entry in the portal vein, decreases mitochondrial-derived reactive oxygen species (ROS) and restores calcium homeostasis, which are both implicated in many neurodegenerative diseases. Due to the established therapeutic benefits of prodrug MP201 in EAE mice, we hypothesized that administration of DNP itself may also have significant potential for therapeutic effects. Here, effects of varying doses of DNP treatment in EAE mice were assessed by the extent of spinal cord paralysis, optokinetic response (OKR), RGC survival, and optic nerve demyelination and inflammation. Results show that daily oral doses of 5-10 mg/kg DNP initiated after onset of EAE can significantly reduce spinal cord paralysis, a marker of the EAE MS-like disease, by day 42 after disease induction. DNP treatment significantly reduces RGC loss induced by optic neuritis in EAE mice; however, effects of DNP do not significantly improve visual function, or optic nerve demyelination and inflammation. Current studies show DNP treatment promotes increased RGC survival, but continued inflammation and demyelination likely reduce visual function, suggesting future studies examining combination therapy of DNP with anti-inflammatory agents may be warranted.

## 1. Introduction

Optic neuritis is an inflammatory demyelinating disease of the optic nerve that often occurs in patients with central nervous system autoimmune demyelinating diseases [1]. Typical cases of optic neuritis, commonly associated with multiple sclerosis (MS), present with unilateral vision loss that has partial improvement over several weeks, but results in some permanent visual loss in about 60 percent of patients [1,2,3]. Permanent visual loss following optic neuritis occurs due to retinal ganglion cell (RGC) damage and axonal loss) [4,5]. The current treatment, corticosteroids, can speed visual recovery, but does not prevent permanent visual dysfunction [1,2,3]. Experimental autoimmune encephalitis (EAE) is an established mouse model for MS. C57/BL6 mice immunized with myelin oligodendrocyte glycoprotein (MOG) exhibit a chronic EAE course which features ascending paralysis involving the tail and hind limbs, due to spinal cord inflammation and secondary demyelination [6]. We and others have shown that optic neuritis also occurs in this EAE model [7,8].

Mitochondrial stress, resulting from reactive oxygen species (ROS), is implicated in vision loss for EAE mice, specifically in the loss of RGCs and their axons, and suppressing mitochondrial oxidative stress has been shown to increase RGC survival in multiple EAE models [9,10,11]. Mitochondrial uncoupling is a process involving the leak of protons from the electron transport system out of mitochondria to generate a membrane potential [12] that is known to decrease reactive oxygen species (ROS) and promote protective effects such as slowing biological aging processes [13]. Uncoupling has also been implicated in neuroprotective strategies. For example, in mice, overexpression of human uncoupling protein 2 (UCP2) promoted cortical neuron survival following stroke and traumatic brain injury (TBI) [14]. Furthermore, overexpression of UCP2 in mice resulted in increased RGC survival following excitotoxicity induced by intraocular injection of either N-methyl-D-aspartate or kainic acid [15]. However, studies investigating the effects of mitochondrial uncoupling on RGC survival in models of optic neuritis are limited, prompting a need for further research.

In previous studies, MP201, the prodrug of 2,4-dinitrophenol (DNP), an uncoupling agent, provided therapeutic benefits in multiple neurodegenerative disease models, including EAE. In a rat model of TBI, MP201 effectively reduced injury-related deficits [16]. In EAE mice, MP201 increased RGC survival and prevented axon loss induced by optic neuritis despite not affecting inflammation [9]. In another study, EAE mice treated with MP201 showed significantly milder paralytic symptoms, reduced demyelination, and decreased axon degeneration in the spinal cord [17]. Administration of DNP itself may also have significant potential for therapeutic effects. In a mouse model of Parkinson’s disease, MP201 preserved dopaminergic neurons and ameliorated motor deficits, and DNP exerted similar effects, but to a lesser extent [18]. In mice, administration of DNP, at 1 mg/L in drinking water, decreased ROS by enacting metabolic processes similar to those resulting from caloric restriction [19]. In a rat model of focal ischemia-reperfusion injury, administration of DNP reduced infarct volume approximately 40% [20]. Furthermore, in a study involving a mouse model of Huntington’s disease, administration of DNP at 1 mg/kg/day resulted in significantly improved motor functions [21]. Most recently, 0.5 mg/kg/day of DNP provided functional recovery from paralysis in the hSOD1G93A model of amyotrophic lateral sclerosis (ALS) [22]. While MP201 has advantages of sustained release driving effects over time, effects of DNP itself suggest it also warrants evaluation, and may offer some advantages over the prodrug. MP201 requires systemic administration to reach the portal vein that contains esterases needed to cleave the prodrug [23], whereas DNP may be suitable for local or topical administration, which could then be explored further if systemic administration first shows significant benefits. In addition, DNP has the potential to follow a faster pathway to clinical use as the FDA has already granted it IND status (#138612), and it is currently in use for a phase I clinical trial in Europe (EUCT number 2023-507195-43-00, euclinicaltrials.eu, Accessed on Jan 7, 2025). In the current study, effects of varying doses of DNP treatment in EAE mice were assessed by the extent of spinal cord paralysis, optokinetic response (OKR), RGC survival, and optic nerve demyelination and inflammation.

## 2. Materials and Methods

### 2.1. Experimental Animals

Female C57/Bl6 mice were obtained from the Jackson Laboratory (Bar Harbor, ME, USA). Mice were housed in an animal facility with a 12-h light/dark cycle at the University of Pennsylvania. All procedures were approved by the Institutional Animal Care and Use Committee at the University of Pennsylvania and completed in accordance with university and federal guidelines.

### 2.2. Induction and Scoring of EAE 

EAE was induced in 8-week-old mice through injection of 200 µg MOG peptide 35-55 (Genscript, Piscataway, NJ, USA) emulsified in Complete Freund’s Adjuvant (Difco, Detroit, MI, USA) and administered in two subcutaneous injections in the back, as in previously published papers [8,9,24]. Control mice were sham-immunized with an equal volume of phosphate buffered saline (PBS) in Complete Freund’s Adjuvant. All mice received intraperitoneal injections of 200 ng pertussis toxin (List Biological, Campbell, CA, USA) in 0.1 mL PBS at 0 h and 48 h post immunization. EAE was scored using a 5-point scale: no disease = 0; partial tail paralysis = 0.5; tail paralysis and waddling gait = 2.0; partial limb paralysis = 2.5; paralysis of one limb = 3.0; paralysis of one limb and partial paralysis of another = 3.5; paralysis of two limbs = 4.0; moribund state = 4.5; and death = 5.0, as previously described [8,9,17].

### 2.3. DNP Treatment 

**Figure 1:**
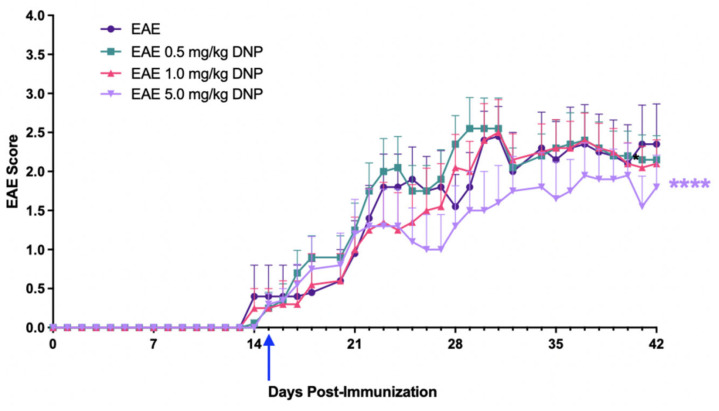
Dose-effects of 0–5.0 mg/kg DNP on EAE scores. DNP was orally administered daily beginning on day 15 (blue arrow) at the indicated doses, and animals were scored for clinical signs of EAE ascending paralysis. Differences among groups of animals were analyzed using a repeated measures one-way ANOVA with Tukey’s multiple comparison test. Mice receiving 5.0 mg/kg DNP had significantly lower EAE scores than mice receiving no DNP (EAE = EAE mice sham-treated with PBS only). **** *p* < 0.0001 (n = 10 mice/treatment).

**Figure 2:**
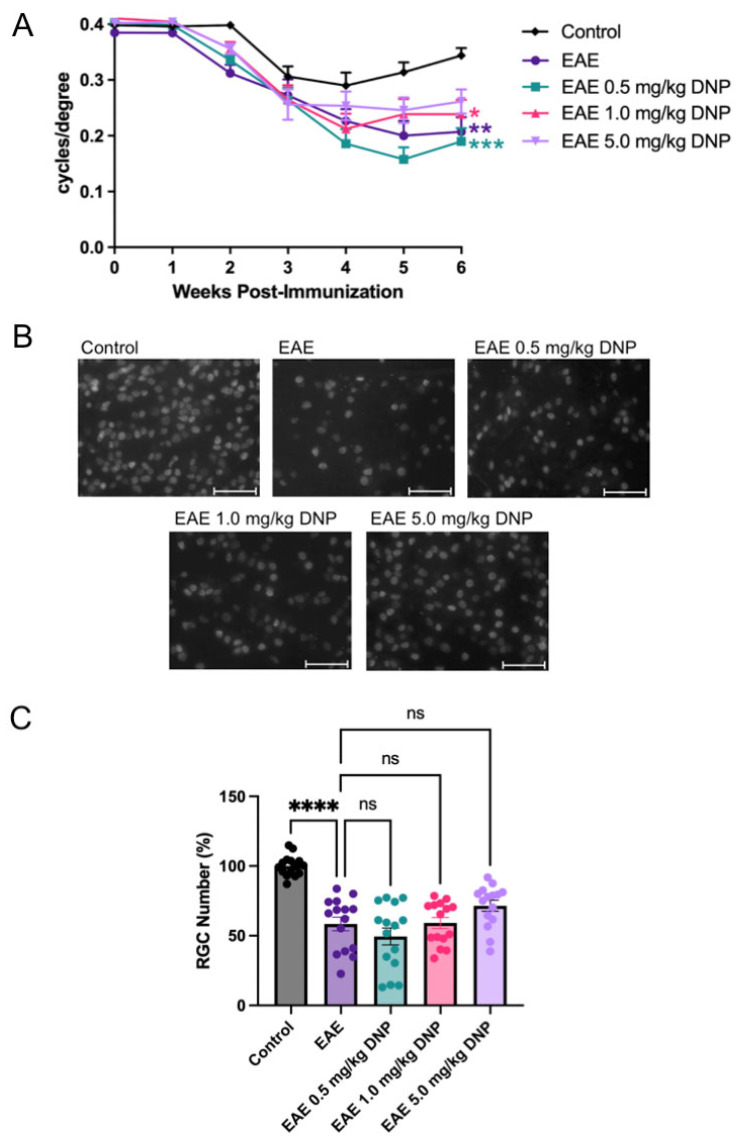
Effects of DNP on visual function and RGC number. (**A**) OKR scores following oral administration of different doses of DNP. Differences among the OKR scores of groups of animals were analyzed using a repeated measures ANOVA with Tukey’s multiple comparison test. Asterisks indicate significant differences as compared with control mice. *** *p* < 0.001, ** *p* < 0.01, * *p* < 0.05 (n = 20 eyes/treatment). There was no significant difference in OKR scores between any of the DNP-treated groups as compared with PBS-treated EAE mice (EAE). (**B**) Representative black and white images of brn3a+ RGCs illustrate the typical density of RGC nuclei (white) that are mainly up to 10 microns in diameter. Scale bars = 50 µm. (**C**) Numbers of RGCs portrayed as percentage of control eyes. Differences among the number of RGCs between groups were analyzed using a one-way ANOVA with Tukey’s multiple comparison test. **** *p* < 0.0001 (n = 15 eyes/treatment), ns = non-significant.

**Figure 3:**
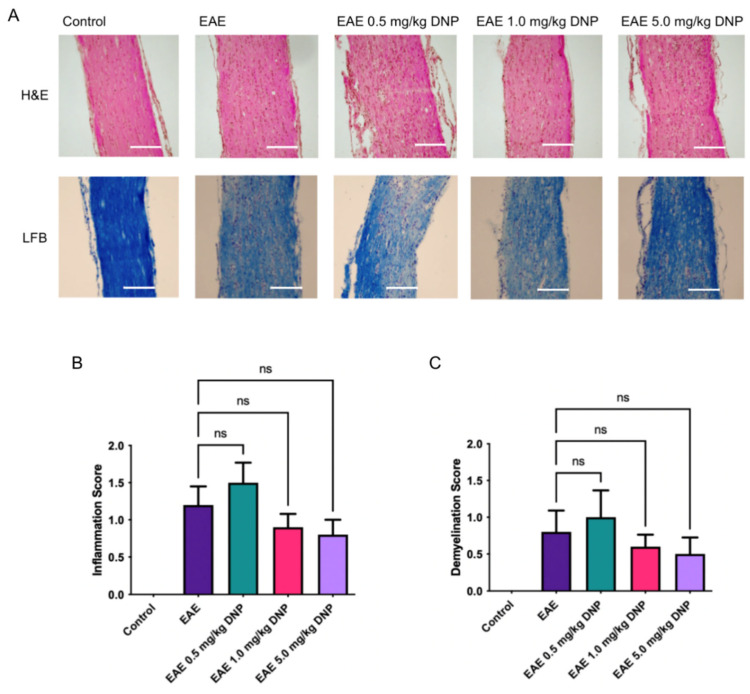
Effects of DNP on optic nerve inflammation and demyelination. (**A**) Representative images of H&E and LFB staining. Scale bar represents 100 µm. (**B**) Inflammation scores following oral administration of DNP show a non-significant trend toward decreased inflammation in optic nerves of EAE mice treated with 1.0 or 5.0 mg/kg DNP. (**C**) Demyelination scores following oral administration of DNP show a non-significant trend toward decreased demyelination in optic nerves of EAE mice treated with 1.0 or 5.0 mg/kg DNP. Thus, DNP treatment, at any dose, did not significantly impact inflammation or demyelination. ns = non-significant.

**Figure 4:**
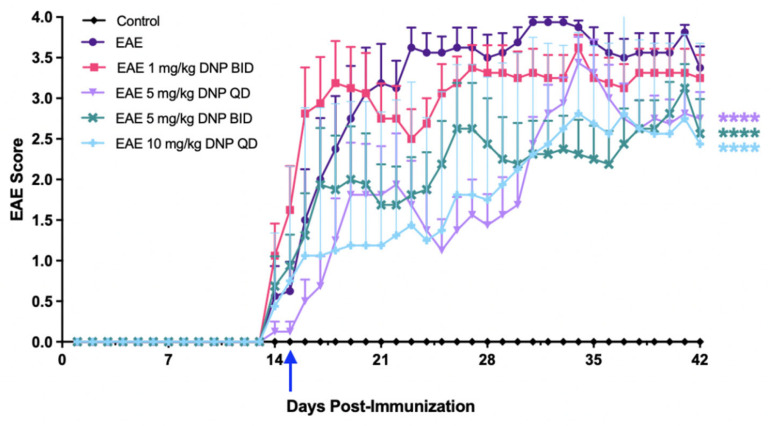
Dose effects of higher DNP doses on EAE scores. Oral administration of DNP, administered daily (QD) or twice daily (BID) as indicated, was initiated 2 weeks after immunization (blue arrow, Day 15), and animals were scored for clinical signs of EAE as described in the Methods section. Differences between groups were analyzed using a repeated measures one-way ANOVA with Tukey’s multiple comparison test. **** *p* < 0.0001 (n = 8 mice/treatment).

**Figure 5:**
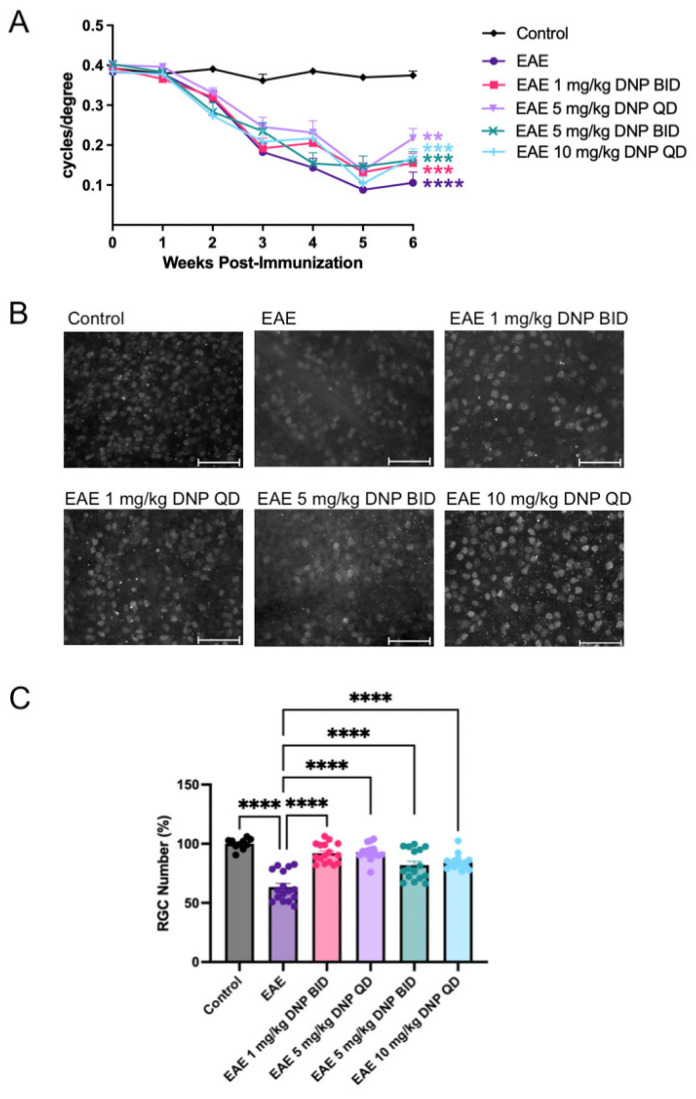
Effects of higher DNP doses on visual function and RGC number. (**A**) OKR scores following oral administration of higher and more frequent doses of DNP. Differences among the OKR scores of groups of animals were analyzed using a repeated measures ANOVA with Tukey’s multiple comparison test. Asterisks indicate significant differences as compared with control mice. **** *p* < 0.0001, *** *p* < 0.001, ** *p* < 0.01 (n = 16 eyes/treatment). There was no significant difference in OKR scores between any of the DNP-treated groups as compared with PBS-treated EAE mice. (**B**) Representative black and white images of brn3a+ RGCs illustrate the typical density of RGC nuclei (white). Scale bars = 50 µm. (**C**) Numbers of RGCs portrayed as percentage of control eyes. Differences among the number of RGCs between groups was analyzed using a one-way ANOVA with Tukey’s multiple comparison test. **** *p* < 0.0001 (n = 16 eyes/treatment).

**Figure 6:**
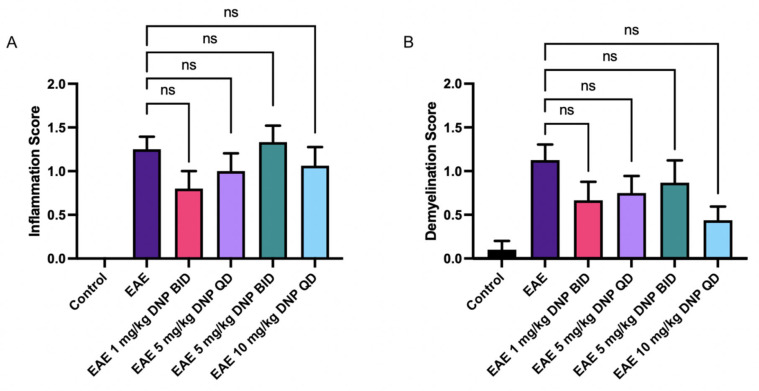
Effects of DNP in high doses on optic nerve inflammation and demyelination. (**A**) Inflammation scores following oral administration of DNP in high doses. (**B**) Demyelination scores following oral administration of DNP in high doses. DNP treatment, at any dose or frequency, did not significantly impact inflammation or demyelination. ns = non-significant.

DNP was dissolved in PBS and administered through oral gavage while control mice received PBS alone. EAE mice were treated by oral gavage once daily (QD) or twice daily (BID) with 0.5, 1.0, 5.0, or 10 mg/kg DNP as indicated, starting from day 15 post immunization until sacrifice (day 42). DNP treatment was administered in two experiments. In the first experiment (Figure 1, Figure 2 and Figure 3), 10 control mice were sham-treated with PBS, and 4 groups of 10 EAE mice/group received once daily 0 (PBS only), 0.5, 1.0, or 5.0 mg/kg DNP. All 20 eyes/treatment group were assessed for OKR responses. Five eyes/group from 5 different mice were randomly pre-selected for protein extraction studies prior to study initiation, and the remaining 15 eyes were used for RGC quantification. In the second experiment (Figure 4, Figure 5 and Figure 6), 8 control mice were sham-treated with PBS, and 5 groups of 8 EAE mice/group received either 0 (PBS only), 5.0, or 10.0 mg/kg DNP daily, or 1.0 or 5.0 DNP twice daily. All 16 eyes were assessed for OKR responses and RGC quantification. 

### 2.4. Measurement of OKR 

To assess visual function, OKR testing was performed. A response reported as cyc/deg was measured as the highest spatial frequency at which mice can track a 100% contrast grating using OptoMetry software and apparatus (Cerebral Mechanics Inc., Medicine Hat, AB, Canada), as in prior studies [8,9,24].

### 2.5. RGC Quantification 

RGCs were immunolabeled in flat-mounted retinas and counted as described previously [8,9]. Retinas were isolated from mice following sacrifice on day 42 and prepared as whole mounts, then permeabilized in 0.5% Triton X-100 in PBS by freezing for 15 min at −70 °C followed by washing in PBS containing 0.5% Triton X-100. Retinas were then incubated overnight at 4 °C with goat-anti-Brn3a antibody (RGC marker) (Santa Cruz Biotechnology, Dallas, TX, USA) diluted 1:100 in PBS containing 2% bovine serum albumin and 2% Triton X-100. The retinas were washed in PBS, incubated at room temperature with Alexa Fluor 488-conjugated anti-goat secondary antibody (Thermo Fisher Scientific, Waltham, MA, USA), diluted 1:500 in blocking buffer, washed in PBS, and mounted on microscope slides in Vectashield mounting medium (Vector Laboratories, Burlingame, CA, USA). RGCs were imaged at 40x magnification in 12 standard fields and counted by a masked investigator using image analysis software as in previous studies [8,9].

### 2.6. Optic Nerve Histochemistry 

In order to evaluate demyelination in the optic nerves, Luxol Fast Blue (LFB) staining was completed. Optic nerve sections were stained with LFB as in prior studies [8,9], and the entire length of each optic nerve section was evaluated by light microscopy by a masked investigator. Demyelination was scored using a 0–3 point relative scale: 0 = no demyelination; 1 = scattered foci of demyelination; 2 = prominent foci of demyelination; and 3 = large (confluent) areas of demyelination. In order to evaluate cellular infiltration as a marker of inflammation in the optic nerves, Hematoxylin and Eosin (H&E) staining was completed. Optic nerve sections were stained with H&E as in prior studies [8,9], and the entire length of each optic nerve section was evaluated by light microscopy by a masked investigator. Inflammation was assessed using a 0–4 point scale: 0 = no infiltration; 1 = mild cellular infiltration of the optic nerve or optic nerve sheath; 2 = moderate infiltration; 3 = severe infiltration; and 4 = massive infiltration. Representative images for both LFB and H&E staining were uniformly enhanced by QuPath to accentuate aspects the masked investigator identified and used for scoring.

### 2.7. Protein Extraction and Western Blot 

Retinas from control and treated groups were homogenized in 200 μl RIPA buffer supplemented with 1x protease inhibitor mixture (Sigma-Aldrich, St. Louis, MO, USA). Protein lysates were centrifuged at 13000 RPM for 5 min and the supernatant was collected and analyzed for protein concentration using BCA quantitative kit. Immunoblotting was performed by standard protocols through fractionation of 25 μg protein lysate by SDS-polyacrylamide gel electrophoresis. Transblot was incubated overnight with primary antibodies (Abcam, Waltham, MA, USA) against PGC-1alpha (1:500 dilution, Abcam) and beta-actin (1:1000). Membranes were incubated for 1 h at room temperature with infrared goat anti-rabbit IgG dye (IRDye 800CW; LI-COR Biosciences–Biotechnology, Lincoln, NE, USA) diluted 1:5000. The intensity of each band was determined using Java-based imaging software (NIH).

### 2.8. Statistical Analysis 

EAE scores and OKR measurements were compared by ANOVA of repeated measures followed by Tukey’s multiple comparisons test, and RGC numbers were compared by one-way ANOVA and Tukey’s multiple comparisons test using GraphPad Prism (GraphPad Software, San Diego, CA, USA). Histologic scores of optic nerve inflammation and demyelination were compared by Kruskal–Wallis Test and Dunn’s multiple comparisons testing using GraphPad Prism. Differences were considered statistically significant at *p* < 0.05. Data are shown as means ± SEM. For optic neuritis experiments, each eye was analyzed as an independent data point similar to prior studies [9]. This is based on prior studies showing that despite a high percentage of eyes developing optic neuritis (about 90%), this does not always directly correlate with the degree of spinal cord disease, and can occur unilaterally or asymmetrically between eyes of the same mouse [8]. Thus, individual eyes are considered independent events. We adjust for these variables by using larger groups of eyes, based on past experience and power calculations, to power studies for each outcome measure.

## 3. Results

### 3.1. Effect of Daily Oral DNP on EAE Scores

We used the mouse model of EAE to determine some of the biological effects of DNP. For this purpose, EAE was induced in 8-week-old female mice. DNP was administered daily through oral gavage beginning two weeks after EAE induction, a time point shown previously to be after onset of vision loss and near the peak of EAE optic nerve and spinal cord inflammation [8]. EAE mice were treated with increasing doses of DNP (0 (PBS only), 0.5, 1.0, or 5.0 mg/kg, respectively). As shown in Figure 1, EAE scores progressively increased, peaked at day 28, and plateaued thereafter. Animals treated with the highest dose of daily DNP (5 mg/kg) showed a significant decrease in EAE score as compared with PBS-treated EAE mice (EAE), suggesting that higher doses of DNP were effective in reducing clinical symptoms of EAE, whereas lower doses (0.5 mg/kg and 1.0 mg/kg) did not significantly reduce EAE scores.

### 3.2. Effects of Daily Oral DNP on Visual Function

Visual function was assessed using OKR. As shown in Figure 2A, OKR scores in PBS-treated EAE mice were significantly reduced compared to control, sham-immunized non-EAE mice. Similarly, OKR scores of DNP-treated EAE mice were significantly reduced compared to untreated control mice, as DNP treatment at 0.5 mg/kg, 1.0 mg/kg, or 5.0 mg/kg did not result in a significant improvement of visual function compared to PBS-treated EAE mice.

### 3.3. Effects of Daily Oral DNP on RGC Survival and Protein Expression

To determine the effect of DNP on RGC number, mice were euthanized 6 weeks after EAE induction. Protein was extracted from 5 eyes/treatment group, and retinal flat mounts from 15 eyes/treatment group were immunostained with the RGC marker, Brn3a. Representative images from PBS-treated non-EAE control, and PBS- and DNP-treated EAE groups are shown in Figure 2B. Immunolabeled RGCs were counted, and the average RGC count per control eye was set at 100 percent. The percentage of RGCs present in the treated and sham-treated EAE mice is shown in Figure 2C. As expected, EAE mice treated with PBS (EAE) showed a 41% decrease in RGC number compared to PBS-treated control mice. EAE mice treated with 5.0 mg/kg DNP showed a small trend toward increased RGC survival, however, this was not significant. Other DNP-treated EAE mouse groups showed similar loss of RGCs, with no significant change compared with PBS-treated EAE mice.

We analyzed the expression pattern of PGC-1alpha protein, a key transcription coactivator that has been implicated as a master regulator of mitochondrial biogenesis and oxidative stress responses in addition to other functions, [25] to explore whether PGC-1alpha may serve as a downstream signal of DNP. Western blot and densitometric analysis of PGC-1alpha protein bands showed no statistically different changes in the levels of PGC-1alpha among all groups (8.97 ± 1.35 for control non-injected, 9.49 ± 1.73 for PBS-treated EAE, 9.08 ± 1.92 for 0.5 mg/kg DNP-treated EAE, 8.66 ± 1.35 for 1.0 mg/kg DNP-treated EAE, and 8.82 ± 0.88 for 5.0 mg/kg DNP-treated EAE).

### 3.4. Effects of Daily Oral DNP on Optic Nerve Inflammation and Demyelination

To determine the effects of DNP on optic nerve inflammation and demyelination, optic nerve sections were stained with H&E and LFB. Representative images shown in Figure 3A were taken from all mouse groups and uniformly enhanced to illustrate the aspects which were used by the masked investigator for evaluation. As shown in Figure 3B,C, DNP treatment had no significant effect on either inflammation or demyelination compared to the PBS-treated EAE mice.

### 3.5. Effect of Higher Doses of DNP on EAE Scores

To determine the effect of DNP in higher, more frequent doses on clinical symptoms of EAE, EAE mice were treated orally with 1.0 mg/kg BID, 5.0 mg/kg QD, 5.0 mg/kg BID, or 10 mg/kg QD DNP. As expected, EAE scores of PBS-treated mice (EAE) progressively increased and peaked following day 28 (Figure 4). EAE mice treated with 5.0 mg/kg either QD or BID, and EAE mice treated with 10 mg/kg QD had significantly lower EAE scores compared to PBS-treated EAE mice.

### 3.6. Effect of Higher Doses of DNP on Visual Function

Visual function was assessed using OKR. As shown in Figure 5A, OKR scores in PBS-treated EAE mice were significantly reduced compared to control (sham-immunized non-EAE) mice. Similarly, OKR scores of DNP-treated EAE mice were significantly decreased compared to untreated control mice. DNP treatment at any dose did not result in a significant increase in visual function compared to PBS-treated EAE mice (Figure 5A).

### 3.7. Effect of Higher Doses of DNP on RGC Number

To determine the effect of DNP in higher, more frequent doses on RGC number, mice were euthanized 6 weeks after EAE induction and retinal flat mounts were immunostained with the RGC marker, Brn3a. Representative images from PBS-treated non-EAE control, and PBS- and DNP-treated EAE groups are shown in Figure 5B. Brn3a-labeled RGCs were counted, and the average RGC count per control eye was set at 100 percent. The percentage of RGCs present in the treated and sham-treated EAE mice is shown in Figure 5C. EAE mice treated with PBS showed a 36% decrease in RGC number compared to control mice. EAE mice treated with all four doses of DNP showed significantly higher RGC numbers compared to PBS-treated EAE mice.

### 3.8. Effect of DNP in Higher Doses on Optic Nerve Inflammation and Demyelination

To determine the effect of DNP in higher, more frequent doses on optic nerve inflammation and demyelination, optic nerve sections were stained with H&E and LFB. As shown in Figure 6A and B, DNP treatment had no significant effect on either inflammation or demyelination, with only non-significant trends as compared with PBS-treated EAE mice.

## 4. Conclusions

C57/BI6 mice inducted with EAE were treated with various doses of DNP and evaluated for EAE symptoms, OKR, RGC survival, and optic nerve inflammation and demyelination. DNP at 5 mg/kg QD or BID and 10 mg/kg QD significantly decreased EAE paralytic scores, and increased RGC survival compared to PBS-treated EAE mice. Interestingly, DNP exerted these neuroprotective effects on RGCs without decreasing optic nerve inflammation or demyelination, and without significantly improving visual function. These results illustrate that DNP has the potential to be an effective treatment at least for the neurodegenerative component of optic neuritis. Results here also confirm prior observations that the degree of optic neuritis may vary in EAE [8] and that the EAE spinal cord disease does not always correlate with the degree of optic neuritis, as our first experiment induced lower EAE scores than our second experiment, but the first experiment induced a larger percentage loss of RGCs. To control for this, each experiment included both a negative (sham-immunized non-EAE mice) and positive (EAE mice sham-treated with PBS) control group to allow internal comparisons with DNP-treatment groups independent of the disease variability often seen in the EAE disease model despite identical disease induction methods [8,9,24].

In previous studies, 5 mg/kg DNP and both mid (16 mg/kg) and high doses (80 mg/kg) of MP201, a prodrug of DNP that provides extended exposure time, was shown to provide therapeutic benefits in EAE mice, including decreased EAE scores and spinal cord inflammation (assessed on day 15 post induction); however, daily treatment was started on day 7, prior to onset of clinical disease [17]. In a prior study, designed closer to the clinical scenario for optic neuritis for when a patient may go to the doctor, MP201 treatment initiated on day 15 after EAE induction increased RGC survival and OKR scores measured at day 42 [9]. In the current study, DNP initiated at the same clinically relevant time point, in doses of 5 mg/kg QD and BID, as well as 10 mg/kg QD, resulted in similarly increased RGC survival and decreased EAE scores. Furthermore, both MP201 and DNP treatment failed to attenuate optic nerve inflammation. However, MP201 treatment attenuated demyelination while treatment with DNP did not attenuate demyelination, suggesting that the prodrug may have superior efficacy with the slower continuous release of DNP and may have additional protective benefits that warrant future exploration.

DNP was first used in munitions production more than 100 years ago, where the workers lost weight by exposure, likely inhalation, and led to a high number of fatalities that decreased after safety measures were implemented [26]. Subsequently, news that DNP can cause weight loss initiated a series of studies at Stanford University in the 1930s, and their publication popularized the use of DNP in over 100,000 humans, albeit at very high doses without knowledge of purity/impurities as a non-FDA-approved weight loss drug without modern-day GLP toxicology studies or manufacturing guidelines, but also with serious risks [27,28]. The risks of these high doses effectively halted pharmaceutical research exploring DNP or uncouplers as potential medications for nearly a century [23]. Despite its tainted complex history, it has been recognized for centuries that dose differentiates toxicity from remedy [29], and now numerous studies have shown that micro-doses of DNP, ~100-300x lower than what was used for obesity, provide potentially disease-modifying therapy for neurodegenerative conditions such as ALS, MS, traumatic brain injury, and Huntington’s, Alzheimer’s, and Parkinson’s Diseases [9,16,17,18,21,22,23,30]. Notably, DNP provided to an ALS model demonstrated recovery from paralysis at, paradoxically, weight-neutral doses (0.5 mg/kg, a human equivalent dose of 2.2 mg/day) [22]. These studies, and others, illustrate that mitochondrial dysfunction is central to the health of the cell, and the pleiotropic pharmacology of DNP delivering a proton (H^+^) to the mitochondrial matrix at micro doses may be pan-neuroprotective.

Overall, prior studies and the current results indicate that MP201 and DNP in low doses both have significant potential to attenuate RGC loss that occurs during optic neuritis. Given that DNP did not decrease optic nerve inflammation, it is possible that ongoing inflammation limits visual function but also suggests that DNP treatment in combination with an anti-inflammatory drug may have significant potential for increased therapeutic benefits. Given that MP201 and DNP each offer distinct advantages (sustained release following systemic administration of MP201, and potential alternative delivery routes and faster clinical translational pathways for DNP), further investigation of both drugs is warranted to understand the potential therapeutic benefits of mitochondrial uncoupling agents in demyelinating disease. 

## Data Availability

The original contributions presented in this study are included in the article. Further inquiries can be directed to the corresponding author.
